# Eukaryotic Initiation Factor 4A-3: A Review of Its Physiological Role and Involvement in Oncogenesis

**DOI:** 10.3389/fonc.2021.712045

**Published:** 2021-08-11

**Authors:** Jiazhou Ye, Xiaomin She, Ziyu Liu, Ziqin He, Xing Gao, Lu Lu, Rong Liang, Yan Lin

**Affiliations:** ^1^Guangxi Medical University Cancer Hospital, Nanning, China; ^2^Guangxi Medical University, Nanning, China

**Keywords:** EIF4A3, cancer, lncRNAs, posttranscriptional regulation, cancer molecular targets and therapeutics

## Abstract

EIF4A3, a member of the DEAD-box protein family, is a nuclear matrix protein and a core component of the exon junction complex (EJC). Under physiological conditions, EIF4A3 participates in post-transcriptional gene regulation by promoting EJC control of precursor mRNA splicing, thus influencing nonsense-mediated mRNA decay. In addition, EIF4A3 maintains the expression of significant selenoproteins, including phospholipid hydroperoxide glutathione peroxidase and thioredoxin reductase 1. Several recent studies have shown that EIF4A3 promotes tumor growth in multiple human cancers such as glioblastoma, hepatocellular carcinoma, pancreatic cancer, and ovarian cancer. Molecular biology studies also showed that EIF4A3 is recruited by long non-coding RNAs to regulate the expression of certain proteins in tumors. However, its tumor-related functions and underlying mechanisms are not well understood. Here, we review the physiological role of EIF4A3 and the potential association between EIF4A3 overexpression and tumorigenesis. We also evaluate the protein’s potential utility as a diagnosis biomarker, therapeutic target, and prognosis indicator, hoping to provide new ideas for future research.

## Introduction

DEAD-box helicases are a family of adenosine triphosphate (ATP)-dependent RNA helicases belonging to the RNA helicase superfamily II. The members of DEAD-box family share nine conserved motifs (Q, Ia, Ib, II, III, IV, V, and VI) (1). DEAD-box helicases were named after the amino acid sequence Asp-Glu-Ala-Asp in motif II. DEAD-box proteins hydrolyze ATP in order to participate in all processes of cellular RNA metabolism such as transcription, mRNA splicing, microRNA processing, and nonsense-mediated mRNA degradation (2).

Eukaryotic initiation factor 4A-3 (EIF4A3), also known as hnRNP265 or DDX48, is a DEAD-box helicase widely distributed in eukaryotes and ubiquitously expressed in the human body (3). EIF4A3 is a nucleocytoplasmic shuttling protein that is found in both the nucleus and the cytoplasm (3–5) and that enters the cytoplasm as part of the exon junction complex (EJC) bound to mRNA. Physiologically, EIF4A3 participates within the splicing-dependent multiprotein EJC to control the splicing of mRNA and monitor mRNA quality before translation, thus regulating RNA metabolism (4, 6). Previous studies have shown that EIF4A3 can bind to several proteins, including the core components of the EJC (7, 8). EIF4A3 is also highly expressed in many tumors, such as glioblastoma, hepatocellular carcinoma (HCC), pancreatic cancer, and ovarian cancer, and it can be recruited by long non-coding RNAs (lncRNAs) to stabilize proteins and promote tumorigenesis. Due to the potential role of EIF4A3 in cancer, it is important to further understand its molecular mechanism and association with tumor development.

## EIF4A3 Structure

**Figure 1:**
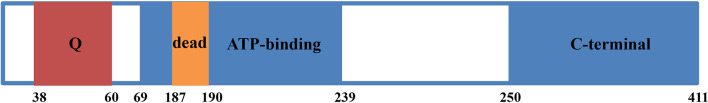
Diagram of EIF4A3 protein structure. EIF4A3 consists of 411 amino acids, with a Q motif at positions 38-60, a DEAD box motif at positions 187-190, a helicase ATP-dependent domain at positions 69-239, and a helicase C-terminal domain at positions 250-411.

EIF4A3 and its predicted gene (KIAA0111) were first identified in 1999 (3). EIF4A3 consists of 411 amino acids, with a DEAD-box motif at positions 187–190 and a Q motif at positions 38-60 (**Figure 1**). EIF4A3 also includes an ATP-dependent helicase motif between amino acids 69–239 and a C-terminal helicase motif between amino acids 250-411 (8). The amino acid sequence of EIF4A3 is similar to that of the human initiation factors EIF4A1 and EIF4A2 (3), while its DEAD-box motif suggested ATP-dependent RNA helicase activity, which opened a new chapter in understanding the role of EIF4A3 in pre-mRNA processing.

## EIF4A3 Is Part of the EJC and Plays a Regulatory Role in mRNA Metabolism

### EIF4A3 Is a Core Component of the EJC

The EJC is a multiprotein complex consisting of four core proteins: EIF4A3, MAGOH (a homolog of Mago-nashi), RBM8A (RNA binding protein 8A, also known as Y14), and CASC3 (also known as metastatic lymph node 51 MLN51, or Barentsz, BTZ) (9). The EJC is deposited by spliceosomes onto mRNAs at 20–24 nucleotides upstream of exon-exon junctions (9), where it serves as a molecular marker for the correct splicing of precursor mRNA. In this way, the EJC contributes to RNA post-transcriptional processes.

**Figure 2:**
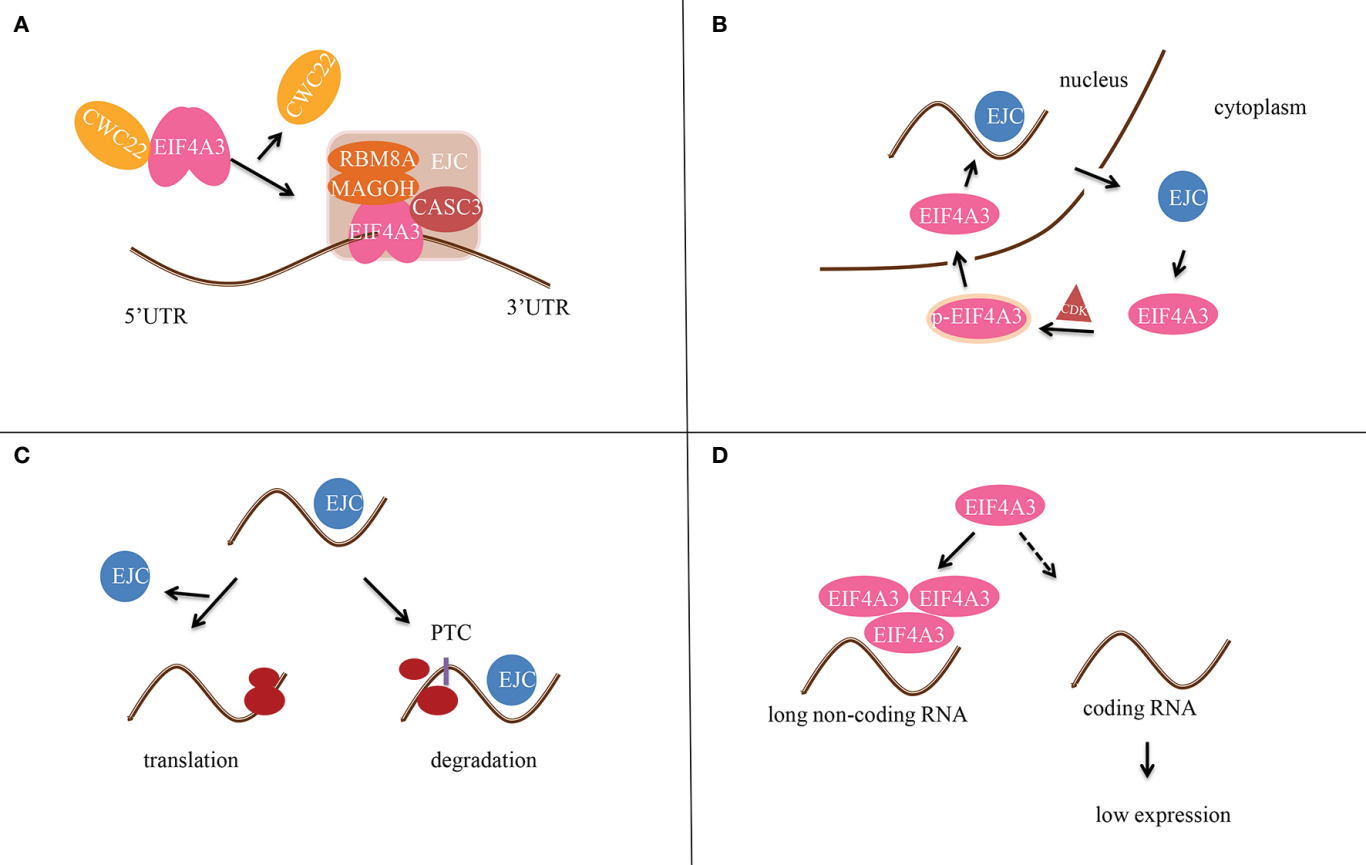
Scheme of the molecular mechanism of EIF4A3 in post-transcriptional gene regulation. **(A)** EIF4A3 first binds to RNA in a CWC22-dependent manner, and then to the MAGOH-RBM8A complex to form the precursor EJC. CASC3 then binds to EIF4A3 after RNA splicing to form the EJC tetramer. **(B)** EIF4A3 is phosphorylated (p-EIF4A3) by CDK1/CDK2 in the cytoplasm and enters the nucleus. After dephosphorylation in the nucleus, the EJC complex is formed. Next, EIF4A3 is transferred out of the nucleus and is released from the EJC complex. **(C)** In the case of normal mRNA, EJC dissociates before translation. In contrast, if the mRNA contains a premature termination codon (PTC), the EJC does not dissociate and it hinders translation. **(D)** EIF4A3 is recruited by long non-coding RNAs (lncRNAs) to reduce its aggregation around RNAs, thereby affecting the translation of target genes.

EIF4A3 was identified as part of the EJC in 2004, when it was found to bind to spliced mRNAs at the same position as the EJC (6). In addition, EIF4A3 binds to mRNA and regulates its splicing by recruiting diverse peripheral proteins, thus affecting subsequent mRNA transportation, localization, translation, and nonsense-mediated mRNA decay (NMD) (**Figure 2**). EIF4A3 also associates with Y14 and MAGOH indirectly by interacting with the mRNA export factors TAP and Aly/REF.

### EIF4A3 Binds to mRNA and Helps Initiate EJC Assembly

EJC assembly is strongly related to mRNA splicing but does not seem to involve RNA sequence specificity. In the EJC, the RecA1 and RecA2 domains of EIF4A3 form sites that can combine with RNA. In addition, EIF4A3 interacts with the phosphate-ribose backbone of RNA through the DEAD-box domain, which prevents the bound RNA from becoming double-stranded. X-ray crystallography has also shown that EIF4A3 switches between a closed and an open conformation. The closed conformation favors binding to ATP and RNA. When EIF4A3 is free or bound only to CASC3, it adopts an open conformation in which the two domains rotate by 160° relative to each other (8), favoring binding of adenosine diphosphate (ADP) and reducing affinity for RNA. Furthermore, the MAGOH-RBM8A heterodimer locks EIF4A3 in the closed conformation by inhibiting EIF4A3 ATPase activity and stabilizing its RNA binding site to avoid RNA dissociation. When the EJC disassembles, MAGOH-RBM8A dissociates, the motif I in EIF4A3 changes its conformation to activate the ATPase, and EIF4A3 dissociates from RNA to adopt an open conformation (8, 10).

The switch in EIF4A3 conformation helps control the initiation of EJC assembly, in which the proteins come together in a specific order. First, EIF4A3 adopts the closed conformation and binds to ATP when complexed with Cef1 (CWC22), a splicing factor whose MIF4G domain directly interacts with the RecA-2 domain of EIF4A3, resulting in spliceosome activation. Then, the MAGOH-RBM8A dimer interacts with EIF4A3 to prevent its conformational change, forming a trimeric pre-EJC, which provides a binding platform for peripheral EJC components such as Aly/REF, SRm160, RNPS1, Pnn, SAP18, and Acinus (11). When the mRNA exons are connected and separated from the EJC, the EJC is released. Finally, CASC3 triggers the formation of tetrameric EJC at the exposed residues 178 and 179 of EIF4A3 (12–15).

Subsequent studies confirmed the function of CWC22 in the EJC and identified the phosphorylation of EIF4A3 in the cytoplasm by cyclin-dependent kinase 1 (CDK1) or 2 (CDK2) as a key step in EJC assembly (16). After phosphorylation, EIF4A3 is transported to the nucleus and binds to CWC22, which then transfers EIF4A3 to the spliceosome, after which CWC22 departs and EIF4A3 binds to other core components of EJC (MAGOH, Y14, and MLN51). Since phosphorylation prevents the binding of EIF4A3 to mRNAs and other EJC components (16), a still unknown phosphatase is likely to dephosphorylate EIF4A3 before EJC assembly.

### EIF4A3 Is Involved in NMD, an RNA Surveillance Pathway

NMD is a quality-control mechanism that is important for RNA splicing and translation in eukaryotes. It ensures the quality and abundance of transcripts by recognizing and degrading abnormal mRNA harboring premature termination codons (PTCs), which could dysregulate numerous biological processes (17, 18). NMD is initiated by a PTC located around 50–55 nucleotides upstream of the EJC, leading to the degradation of mRNAs encoding truncated proteins (19). NMD has also been documented during the processing of various spliced mRNAs, indicating its vital role in mRNA regulation (20).

The EJC was first discovered as a mammalian NMD regulator (21, 22), and EIF4A3 proved to be fundamental for activating the NMD factors UpF1, Upf2, and Upf3b (23). EIF4A3 depletion was found to increase the level of mRNAs containing PTCs and to weaken NMD (24, 25). In fact, downregulation of EIF4A3 upregulated Arc, whose expression is normally regulated through NMD (23).

The cell cycle-dependent phosphorylation of EIF4A3 at threonine 163 (T163), which takes place in the late M phase, has also recently been reported to contribute to NMD inhibition when the cycle of EJC assembly–disassembly is blocked (16). These results were supported by the delayed M-to-G1 progression in cells expressing the T163D mutant of EIF4A3, which indicated that NMD efficiency and the M-to-G1 transition can be restored by EIF4A3 dephosphorylation *via* a still unknown phosphatase.

### EIF4A3 Acts as a Transcript-Selective Translational Repressor of Selenoprotein Synthesis During Selenium Deficiency

Selenoproteins are proteins containing selenocysteine (Sec) encoded by the UGA codon. Incorporation of Sec into a growing polypeptide chain requires a Sec insertion sequence (SECIS) in the 3’-untranslated region of the transcript (26, 27). EIF4A3 can regulate selenoprotein translation by interacting with SECIS (28). This interaction requires two globular domains of EIF4A3 as well as the internal and apical loops of SECIS. The binding of EIF4A3 to SECIS blocks the normal SECIS binding protein 2-mediated mechanism of Sec insertion, thus reducing selenoprotein synthesis. EIF4A3 can also selectively regulate selenoprotein expression in response to selenium. In particular, during selenium deficiency, EIF4A3 is upregulated, potentially at the post-transcriptional level, but the exact mechanism is unknown. The protein binds to SECIS in the mRNA encoding glutathione peroxidase 1, a dispensable selenoprotein, thereby reducing its synthesis (29). In contrast, EIF4A3 has low affinity for mRNAs encoding essential selenoproteins, such as phospholipid hydroperoxide glutathione peroxidase and thioredoxin reductase, such that their expression remains relatively constant (28, 29). Hence, by regulating the translation of selenoproteins, EIF4A3 supports necessary physiological activities under selenium-deficient conditions.

## EIF4A3 Acts as an Oncogene in Malignant Tumors

Recent studies have shown that EIF4A3 is significantly upregulated in several malignant tumors. In most cases, the interaction between EIF4A3 and lncRNAs plays a vital role in oncogenesis by participating in the post-transcriptional regulation of RNA, thereby affecting gene expression (**Table 1**). LncRNAs are non-coding RNAs with more than 200 base pairs, including long intergenic non-coding RNAs (lincRNAs), antisense lncRNAs, pseudogenes, and circular RNAs. In recent years, it has been shown that lncRNAs may affect gene expression by acting as competitive endogenous RNAs (ceRNAs). MicroRNAs (miRNAs) are non-coding single-stranded RNAs that bind to miRNA response elements (MREs) on the target mRNA to block its translation. Recent evidence suggests that ceRNAs compete with miRNAs for binding to MREs, thus preventing miRNA-mediated downregulation (44–46).

**Table 1:** Proposed roles of EIF4A3 in tumors.

Tumor type	Mechanism	References
Glioblastoma	EIF4A3 can stabilize LINC00680 and TTNAS1, induce cyclization of circMMP9 and increase its expression, thus promoting tumor proliferation, migration, and invasion	(30, 31)
Hepatocellular carcinoma	EIF4A3 is related to splicing, chemokine signaling, and cell cycle	(32, 33)
Pancreatic cancer	EIF4A3 is recruited by LICN01232 to stabilize TM9SF2 mRNA	(34, 35)
Ovarian cancer	Some anti-ovarian drugs target the axis linking the lncRNA CASC2 and EIF4A3	(36, 37)
Colorectal cancer	EIF4A3 binds to H19, affecting the expression of cell cycle regulatory genes. EIF4A3 also acts as a downstream target of the ZFAS1-NOP58-SNORD12c/78 signaling pathway	(38, 39)
Gastric cancer	EIF4A3 promotes cyclization of HSA_circ_001988, and acts as a mediator of VCAN-AS1 to regulate the expression of TP53	(40, 41)
Triple-negative breast cancer	EIF4A3 regulates the synthesis and expression of circSEPT9	(42)
Non-small cell lung cancer	EIF4A3 shortens the half-life of VEGFA mRNA	(43)

Further studies on EIF4A3-related tumor molecular mechanisms revealed an important role of EIF4A3 in tumor occurrence, development, and metastasis. Thus, EIF4A3 has potential value in clinical applications as a prognostic marker or therapeutic target, which may provide a new direction in cancer research and treatment.

### EIF4A3 in Glioblastoma

EIF4A3 is overexpressed in glioblastoma and is related to two lncRNAs, LINC00680 and TTNAS1, that promote the malignant behavior of glioblastoma cells (30). That study showed that when the expression of EIF4A3, LINC00680, and TTNAS1 was downregulated in PANC-1 and SW1990 cells, their proliferation, migration, and invasion was impaired, while tumor growth was inhibited *in vivo* and glioblastoma cell apoptosis was promoted. EIF4A3 downregulation also shortened the half-lives of LINC00680 and TTN-AS1. These results indicate that EIF4A3 may stabilize LINC00680 and TTNAS1, prolonging the half-life of these lncRNAs and providing an important potential therapeutic target for glioblastoma treatment.

Another study in glioblastoma showed that EIF4A3 can regulate the expression of hsa_circ_0001162 (circMMP9) by binding to an upstream mRNA region (31). EIF4A3-induced circMMP9 is a circular RNA involved in glioblastoma cell proliferation, invasion, and metastasis. RNA immunoprecipitation and RNA pull-down assays identified the binding sequence of EIF4A3 in the upstream region of circMMP9. Furthermore, EIF4A3 overexpression upregulated circMMP9, while EIF4A3 underexpression had the opposite effect. Thus, EIF4A3 may induce the formation of circMMP9 by binding to circMMP9 mRNA, increasing the expression of circMMP5 in glioblastoma and promoting tumor progression.

Based on these studies, circMM9 is a ceRNA that targets miR-124, while LINC00680 and TTNAS1 are ceRNAs that target miR-320b in glioblastoma cells. In addition, these findings suggest that the tumor-promoting role of EIF4A3 in glioblastoma may be related to the upregulation of ceRNAs.

### EIF4A3 in HCC

The phosphorylation of splicing proteins such as EIF4A3 has been investigated in the HCC metastatic cell line MHCC97-H-1 (32). That work linked such phosphorylation to upregulation of genes involved in transcriptional regulation, mRNA processing, RNA splicing, the spliceosome, insulin signaling pathway, and the cell cycle in HCC. These results suggest that EIF4A3 may modulate mRNA biological functions through phosphorylation and may thereby participate in the development of HCC.

A recent study also found EIF4A3 overexpression in HCC tissue data from The Cancer Genome Atlas (TCGA) database, which was associated with poor prognosis (33). This observation was validated in human tissues, where several chemokine signaling pathways that affect HCC by regulating inflammation were linked to genes that are also involved in EIF4A3 upregulation. Genes positively correlated with EIF4A3 were tumor-related genes. Protein–protein interaction network analysis also suggested that EIF4A3 is associated with the splicing complex, ribosome, and cell cycle in HCC. EIF4A3 may also bind directly to exon regions of the cell-cycle regulatory genes CDK1 and CDK2, CHEK1, and E2F1, possibly regulating their expression. In that study, EIF4A3 was concluded to be related to RNA splicing, chemokine signaling, and the cell cycle, suggesting that EIF4A3 acts as a bridging protein in HCC, making it a potential therapeutic target.

### EIF4A3 and Pancreatic Cancer

EIF4A3 antigen and antibodies might be useful serum markers in pancreatic cancer diagnosis (34), as they are more strongly expressed in most pancreatic cancer tissues than in normal tissues. Another study found that EIF4A3 is recruited by LINC01232 to stabilize transmembrane 9 superfamily member 2 (TM9SF2) mRNA and regulate its expression, thereby contributing to pancreatic adenocarcinoma (PAAD) (35). Analysis of the TCGA database and clinical tissues showed that the expression of LINC01232, TM9SF2, and EIF4A3 is upregulated in PAAD. LINC01232 is an lncRNA that is highly expressed in PAAD and has been associated with poor prognosis (47). However, no correlation between EIF4A3 and LINC01232 expression has been identified, suggesting that EIF4A3 might be recruited by LINC01232. Further experiments showed that LINC01232 reduced the stability of TM9SF2 mRNA, while EIF4A3 upregulation had the opposite effect. These results suggest that EIF4A3 may contribute to PAAD progression by stabilizing TM9SF2 mRNA.

### EIF4A3 and Ovarian Cancer

Studies of EIF4A3-related ovarian cancer have focused on the molecular mechanisms of antitumor drugs. That work has shown that sanguinarine and ivermectin can regulate the lncRNA-EIF4A3-mRNA axis, suggesting that they might have anti-ovarian cancer activity and that EIF4A3 may participate in the progression of ovarian cancer (36, 48).

Sanguinarine has been found to inhibit tumor cell viability and promote apoptosis by inducing the expression of lncRNA cancer susceptibility 2 (CASC2) (48). EIF4A3 was also identified as a CASC2-binding protein. Downregulation of CASC2 reversed the suppression of sanguinarine-induced tumor migration and invasion. However, downregulation of EIF4A3 reversed the effect of CASC2 reduction when combined with sanguinarine in ovarian tumor cells, while it also reversed the effect of CASC2 on the NF-κB and PI3K/AKT/mTOR pathways. These results suggest that EIF4A3 is associated with ovarian cancer, and that its interaction with CASC2 affects the antitumor activity of drugs.

The lncRNA SNHG3 was also identified as a mitochondrial differential expression protein and potential biomarker in epithelial ovarian cancer. Its ability to regulate energy metabolism appears to be influenced by EIF4A3 and miRNAs (49).

Quantitative proteomics analysis showed that ivermectin can suppress EIF4A3 and 116 EIF4A3-associated proteins in ovarian cancer (36). In addition, 16 lncRNAs have been associated with ovarian cancer, and the combination of three of them into a tumor prognosis model showed high prognostic value (50).

In summary, the binding of EIF4A3 to lncRNAs inhibits the ability of EIF4A3 to target mRNAs, thus favoring ovarian cancer progression. Thus, the lncRNA-EIF4A3-mRNA axis may be an important pathway for suppressing ovarian cancer growth.

### EIF4A3 and Other Tumors

The lncRNA H19 has been identified as a tumor-promoting factor in colorectal cancer (CRC), upregulating a series of cell-cycle regulatory genes, such as cyclin D1, cyclin E1, and CDK 4, thus promoting CRC cell proliferation (38). Further experiments confirmed that EIF4A3 binds to H19, blocking the recruitment of EIF4A3 to cell-cycle regulatory genes, which in turn increases the expression of cell-cycle regulatory genes, accelerates cell cycle progression, and promotes CRC. The ZFAS1-NOP58-SNORD12C/78 pathway is another known key regulator in CRC (39), and co-expression analysis showed that both EIF4A3 and LAMC2 might be the downstream target genes of this pathway. This hypothesis was supported by the observation that SNORD12C/78 downregulation significantly reduced the expression of EIF4A3 and LAMC2 by affecting their RNA half-life. These results suggest that ZFAS1-NOP58-SNORD12C/78 might promote tumor cell proliferation and inhibit tumor cell apoptosis by affecting the expression of downstream target genes such as EIF4A3 and LAMC2.

In addition to its indirect association with gastric cancer, EIF4A3 appears to act as a regulatory factor of other key genes related to cancer progression. One study found that Hsa_circ_001988 suppressed proliferation and invasion of gastric cancer, and that EIF4A3 might promote the cyclization of Hsa_circ_001988 (40). In another study, EIF4A3 served as a bridge for lncRNA VCAN antisense RNA 1 (VCAN-AS1), which modulates TP53 expression (41). Specifically, VCAN-AS1 may competitively bind to EIF4A3, hamper the binding of EJC to spliced mRNPs containing TP53 mRNA, and hinder p53 expression, thereby promoting progression of gastric cancer.

EIF4A3 has also been related to other tumors. The protein circSEPT9 promotes proliferation and invasion of triple-negative breast cancer (43). EIF4A3 can bind to upstream and downstream regions of circSEPT9 precursor mRNA and promote its expression (42). In addition, EIF4A3 is overexpressed in non-small cell lung cancer (NSCLC), where it competitively binds to LINC00667 and thereby upregulates expression of its target gene encoding vascular endothelial growth factor A (VEGFA). EIF4A3 inhibition not only reduces tumor proliferation, migration, and angiogenesis, but it also shortens the half-life of VEGFA, suggesting that LINC00667-VEGFA-EIF4A3 is an oncogenic pathway in NSCLC.

## EIF4A3 as a Therapeutic Target

The presence of a PTC in mRNA can lead to a large number of genetic and sporadic diseases. However, NMD can control the quality of mRNA by eliminating PTC-containing mRNA (17, 51). Therefore, NMD inhibitors are considered a novel treatment strategy against various diseases (52). Since EIF4A3 plays a key role in NMD, EIF4A3-targeted strategies may open a new path for treating cancer and other PTC-related genetic diseases.

Pateamine A has been identified as a small-molecule inhibitor of EIF4A, which can stabilize the complex of EIF4A and targeted RNA, and prevent the formation of the EIF4F complex, thus inhibiting the initiation of eukaryotic translation and cell proliferation (53). Hippuristanol, a natural product from marine plants, was also reported to inhibit EIF4A, blocking the proliferation of human T lymphotropic virus type 1-infected T-cells and adult leukemia T-cells by inducing cell apoptosis and cell cycle arrest during G1 phase. However, hippuristanol had no effect on peripheral blood mononuclear cells (54). More recently, high-throughput screening identified a class of 1,4-diacylpiperazine derivates as promising EIF4A3 allosteric inhibitors, and an EIF4A3 inhibitor that could bind to the allosteric site of EIF4A3 was discovered, with an IC_50_ of 0.11 µM (55). Further optimization of this analogue led to the first ATP-competitive EIF4A3 inhibitor with excellent ATPase inhibitory activity (55). Additional chemical optimization afforded two 1,4-diacylpiperazine derivates with IC_50_ values of 0.20 and 0.26 µM, which inhibited EIF4A3 and cellular NMD with high selectivity (56). Another inhibitor of both EIF4A3 and DEAD-box helicase 3 was identified using high-throughput RNA helicase assays based on fluorescence resonance energy transfer. In that assay, the ability of compounds to inhibit ATPase activity correlated with their ability to inhibit helicase activity (57).

Traditional tumor-targeted therapy focuses on antigens expressed on tumor cells, but tumor cells can downregulate such antigens. In contrast, inhibiting EIF4A3 may lead to the production of new antigenic determinants. Further studies are needed to develop more effective EIF4A3 inhibitors and apply them to treatment of cancers and other PTC-related diseases.

## Discussion

EIF4A3, a core component of the EJC, regulates RNA metabolism by participating in RNA splicing and affecting its downstream events. Recent studies have shown that EIF4A3 is overexpressed in various tumors, such as glioblastoma, HCC, PAAD, and ovarian cancer, where the interaction between lncRNAs and EIF4A3 plays an important oncogenic role. LncRNAs may modulate tumor gene expression by affecting the recruitment of EIF4A3 to RNA, EJC assembly, RNA splicing, and other downstream events. Recent research has also shown that EIF4A3 may have potential value in tumor diagnosis, treatment, and prognosis.

Nevertheless, much remains to be clarified about EIF4A3 function in light of the complexity of gene expression regulation as well as the onset and progression of cancer. For instance, EIF4A3 and other phosphoproteins in the spliceosome are considered to be involved in the occurrence and development of HCC by regulating the physiological functions of mRNAs (33). However, EIF4A3 phosphorylation in other tumors, such as renal cancer and malignant hematological diseases, has not been investigated, while the signaling pathways in HCC, breast cancer, lung cancer, and other tumors are poorly understood. Therefore, additional studies are needed to understand the mechanism of EIF4A3 and its role in different tumors. Moreover, the correlation of EIF4A3 with lncRNAs, and the underlying mechanisms involved, have not been widely studied. Although highly selective EIF4A3 inhibitors have been developed to examine the role of EIF4A3 in disease, the development of drugs targeting EIF4A3 remains in the preclinical stage. Further research is needed to clarify the role of EIF4A3 in tumor development and identify its potential clinical applications as a diagnostic marker or therapeutic target.

## Author Contributions

All the authors contributed to the preparation of this work. JY and XS drafted and revised the article. ZL and ZH were responsible for the topics, final editing, and preparation of the manuscript for submission. XG, LL, RL, and YL critically revised the manuscript. All authors contributed to the article and approved the submitted version.

## Funding

This research was supported by the National Natural Science Foundation of China (81803007, 82060427), Guangxi Key Research and Development Plan (GUIKEAB19245002), Guangxi Scholarship Fund of Guangxi Education Department, General Program of Guangxi Natural Science Foundation (2020GXNSFAA259080), Youth Talent Fund Project of Guangxi Natural Science Foundation (2018GXNSFBA281030, 2018GXNSFBA281091), Guangxi Medical and Health Appropriate Technology Development and Application Project (S2017101, S2018062), Guangxi Medical University Training Program for Distinguished Young Scholars, and Science and Technology Plan Project of Qingxiu District, Nanning (2020037, 2020038).

## Conflict of Interest

The authors declare that the research was conducted in the absence of any commercial or financial relationships that could be construed as a potential conflict of interest.

## Publisher’s Note

All claims expressed in this article are solely those of the authors and do not necessarily represent those of their affiliated organizations, or those of the publisher, the editors and the reviewers. Any product that may be evaluated in this article, or claim that may be made by its manufacturer, is not guaranteed or endorsed by the publisher.

## Abbreviations

EJC, exon junction complex; NMD, nonsense-mediated mRNA decay; ATP, adenosine triphosphate; HCC, hepatocellular carcinoma; lncRNA, long non-coding RNA; ADP, adenosine diphosphate; RBM8A, RNA binding protein 8A; CDK, cyclin-dependent kinase; PTC, premature termination codon; Sec, selenocysteine; SECIS, Sec insertion sequence; lincRNA, long intergenic non-coding RNA; ceRNA, competitive endogenous RNA; miRNA, microRNA; MRE, miRNA response element; TCGA, The Cancer Genome Atlas; PAAD, pancreatic adenocarcinoma; TM9SF2, transmembrane 9 superfamily member 2; CASC2, cancer susceptibility 2; CRC, colorectal cancer; VCAN-AS1, VCAN antisense RNA 1; NSCLC, non-small cell lung cancer; VEGFA, vascular endothelial growth factor A.
